# Dose-Response Relationships between Training Load Measures and Physical Fitness in Professional Soccer Players

**DOI:** 10.3390/ijerph18084321

**Published:** 2021-04-19

**Authors:** Saeid Younesi, Alireza Rabbani, Filipe Manuel Clemente, Rui Silva, Hugo Sarmento, António José Figueiredo

**Affiliations:** 1 University of Coimbra, Research Unit for Sport and Physical Activity, Faculty of Sport Sciences and Physical Education, 3004-531 Coimbra, Portugal; saeidyounesi78@yahoo.com (S.Y.); hg.sarmento@gmail.com (H.S.); afigueiredo@fcdef.uc.pt (A.J.F.); 2 Department of Exercise Physiology, Faculty of Sport Sciences, University of Isfahan, Isfahan 8174673441, Iran; alireza.rabbani@gmail.com; 3 Instituto Politécnico de Viana do Castelo, Escola Superior Desporto e Lazer, Rua Escola Industrial e Comercial de Nun’Álvares, 4900-347 Viana do Castelo, Portugal; ruimiguelfps@hotmail.com; 4 Instituto de Telecomunicações, Delegação da Covilhã, 1049-001 Lisboa, Portugal

**Keywords:** football, performance, athletic performance, sports training, internal load, external load

## Abstract

The aim of this cohort study was two-fold: (i) to analyze within-group changes of final velocity in a 30-15 intermittent fitness test (V_IFT_), final velocity in a Vameval test (V_vameval_), 20-m sprint and countermovement jump (CMJ); (ii) to explore the relationships between V_IFT_ and Vvameval outcomes and their changes with internal and external loads. Twenty-two professional soccer players (mean ± SD; age 27.2 ± 3.4 years, height 174.2 ± 3.6 cm, body mass 69.1 ± 6.4 kg, and body fat 10.4 ± 4.1%, 3.1 ± 1.5 years in the club) participated in this study. External and internal loads were obtained using global positioning system, heart rate and rate of perceived effort (sRPE) after each training session. Players were assessed in CMJ, 20-m sprint, Vameval and 30-15 intermittent fitness test, before and after the observed period. Very large relationships were observed between V_IFT_ and Vameval for pre- (*r* = 0.76), post (*r* = 0.80) and pooled-data (*r* = 0.81). V_vameval_ showed less sensitivity (−22.4%, [−45.0 to 9.4]), ES −0.45 [−1.05 to 0.16]) than V_IFT_. ∆V_IFT_ had unclear associations with all sRPE, but had moderate correlations with objective internal and external measures, while, ∆V_vameval_ varied between large and very large relationships with all sRPE, but had unclear associations with all other selected training loads. Objective internal and external loads may be used to track aerobic power related changes from V_IFT_.

## 1. Introduction

In sports context, dose-response relationships are simply referred to the magnitude of a biological response, depending on the exposure to a given training stimulus after a certain time-period [1]. In that sense, it has been suggested that chronic exposures to training loads are associated with more resilient athletes that are capable of sustaining greater acute training loads, without higher injury risks [2,3]. Despite that, the principles of training such as individualization, progression and overload, must be carefully followed for ensuring that athlete’s dose-responses are adequate and have the desired effects on them [4]. Considering that in professional soccer teams, coaches typically follow a one-size-fits-all soccer practice, the above-mentioned training principles may not be adequately imposed [5].

The dose-response relationship is highly dependent on different determinants of player’s physical fitness levels, as the same stimuli might be perceived higher or lower for one athlete, and not for others. Given that, the response of a given dose is certainly different between athletes, resulting in within-team variations [6]. Usually, training is quantified through different methods, depending on teams’ different budgets. For instance, the external loads (imposed stimulus) are commonly quantified via global position systems (GPS) in soccer [7]. The internal loads (response to a given imposed stimulus) can be quantified via objective measures such as heart rate (HR), and biochemical markers [8,9]. On the other hand, internal loads can also be quantified via subjective measures, such as session-rate of perceived exertion (sRPE), and perceived level of exertion for respiratory and leg musculature efforts [10,11], and its associated indices [12]. Both, internal and external load measures have been consistently researched for their meaningful associations, although providing different information for sports scientists and coaches [8,9].

The imposed training loads may be perceived differently between players and produce different adaptations. For those reasons, it is of paramount importance to establish associations between internal (objective and subjective) and external training load measures with the different determinants of player’s physical fitness responses. In fact, considering the subjective measures of internal load quantification and its relationships with possible performance changes during intermittent-based field tests, such as the 30-15 intermittent fitness test (30-15IFT), it was previously revealed strong associations exist between sRPE and VIFT measure [13].

Moreover, negative associations between cumulative lower limb perceived load exertions and countermovement jump performance changes has been documented [9]. Considering the objective internal load measures such as HR variables, contradictory evidence has been documented [13,14]. Despite that, strong associations between HR measures (training impulse, TRIMP) and aerobic performance, as well as field tests performance improvements has been revealed [14]. Also, considering the different commonly used TRIMP methods, it was revealed that despite the Bannister’s TRIMP method significantly correlated with sRPE, it was not associated to aerobic fitness adaptations [15]. 

Furthermore, quantifying training and match activity is now facilitated by the use of GPS systems that enables coaches to extract information about weekly distance- and accelerometry-based measures [16,17]. Given that, using these metrics to analyze their dose-relationships with physical fitness of soccer players is a topic of interest. However, there is a lack of studies supporting these associations [8,18,19].

In fact, one of the few studies that analyzed external load dose-response relationships, found large associations between the weekly time spent above maximal aerobic speed (MAS) and adaptations in aerobic fitness [8]. However, unclear correlations were found between different high-intensity running thresholds and MAS [8]. Similarly, a study conducted on eleven professional soccer players, revealed unclear relationships between very high-intensity running, total distance and changes in V_IFT_ [19]. Interestingly, the same study [19], revealed large relationships between accumulated new body load (NBL) and aerobic changes. Also, a similar accelerometry metric (player load), had demonstrated associations with variations in different fitness determinants [18].

As mentioned earlier, there are conflicting evidence surrounding the dose-response relationships between internal loads and physical fitness changes, and there are still a lack of studies analyzing dose-response relationships using different external load measures in adult professional soccer players. For instance, one of the studies that analyzed external loads dose-response relationships used a sample of amateur soccer players [18], while another study used a sample of youth soccer players [8]. Moreover, only one study, to the best of our knowledge, was conducted on professional adult soccer players [19], but only testing the effects on 30-15IFT and not in other determinants of performance as sprinting or vertical jump.

As the training process may be reflected by a highly within- and between-players variation in terms of responses to the imposed loads, it is of interest to understand the relationships between both internal (objective and subjective) loads and external (distance-based and accelerometry-based) loads with possible changes in different levels of fitness parameters. Despite some studies testing the effects of specific load parameters in fitness changes, no study has been included both internal and external load demands, and also analyzed relationships with different fitness tests that are tested for their relationship (e.g., 30-IFT and Vameval). This can be interesting, particularly to identify how tests can be related in their changes, and how load can be associated with that. For those reasons, the purposes of the present study were: (i) to analyze the within-group changes of V_IFT_, Vameval, 20-m sprint and CMJ; and (ii) to explore the relationships between V_IFT_ and Vameval tests as well as their changes (i.e., ∆V_IFT_ and ∆Vameval) with accumulated training load indices. We hypothesize that beneficial changes will occur after the cohort in the fitness performance, while sRPE will be the measure with a better dose-response relationship since represents both dimensions of load (internal and external).

## 2. Materials and Methods

### 2.1. Experimental Approach to the Problem

An observational analytic cohort design was implemented in this study. First fitness assessments of the players were conducted one week prior to the beginning of 2018/2019 pre-season, between 24th June and 1st July and second test was performed immediately after preparation phase between 19 and 26 August. All the materials were the same and the environmental conditions were almost similar during fitness assessments (indoor track, ambient temperature and relative humidity, ranging between 22 and 26 °C and 45 and 52% respectively). For each assessment, temperature and relative humidity were collected two times, and the mean value was registered. However ambient temperature and relative humidity varied greatly over the training data collection phase, ranging between 25 and 32 °C and 55 and 76%, respectively). For each training, temperature and relative humidity were collected two times, and the mean value was registered. Training intervention was implemented from 2 July to 18 August. Training time in the morning and afternoon were between 10:00–12:00 and 17:00–20:00, respectively. During training intervention phase, which lasted 47 days, the external and internal loads were obtained using global positioning and HR monitoring systems, respectively. The sRPE was also collected after each training session. All players involved in the study were professional and were familiar with the GPS system and sRPE methods.

### 2.2. Participants

Twenty-two professional soccer players (mean ± SD; age 27.2 ± 3.4 years, height 174.2 ± 3.6 cm, body mass 69.1 ± 6.4 kg, and body fat 10.4 ± 4.1%, 3.1 ± 1.5 years in the club), all members of a professional club competing in the 2018/2019 season of Qatar Stars League (Qatar First Division), participated in this study. Sample was chosen in convenience, as well as the sample size. The inclusion criteria were (i) participation in all assessments and training sessions, (ii) absence of injuries, physical constrains, or illnesses exhibited during sessions occurred in the period and two weeks prior to the data collections; and (iii) absence of signals of fatigue on assessment days. Players were daily monitored for the training load parameters; thus, the follow-up was ensured by daily collecting information from the players. None of the included players had an illness or chronic clinical conditions, all of them were professional and fully dedicated to the team. All players were informed of the experimental procedures and related risks and gave informed consent before commencing the study. The study protocol was approved by the Scientific Committee of School of Sport and Leisure (Melgaço, Portugal) with the code number CTC-ESDL-CE00118. The study followed the ethical standards of the Declaration of Helsinki.

### 2.3. Fitness Assessment

Assessments included anthropometric assessments conducted on the first day. On the following day, the players were evaluated in countermovement jump, 20-m sprinting test, followed by Vameval test. The 30-15IFT was performed three days after. Training intervention included 47 days (morning or evening sessions) including five friendly matches, six days off and six recovery sessions. To avoid bias in data collection, the players were familiarized with the testing protocols and the instruments of load monitoring were individualized. Additionally, the observers during fitness assessment were blind to the study protocol to minimize the risk of bias. Aiming to minimize the effects of confounders variables, before assessment periods the players had rest for 48-h and had similar patterns of dietary and supplementation and sleep routines.

#### 2.3.1. The 30–15 Intermittent Fitness Test

The 30-15IFT consisted of 30 s of running interspersed by 15 s of walking for recovery. Players were required to run between two lines positioned 40-m apart and return. The test started at 8 km·h^−1^ followed by 0.5 k/h increments every 30 s. At every 30 s, a beep sounded to signal the start of the 15 s of recovery. During the 15 s of recovery the athletes had to stay within the 3-m limits outlined between each line of cones and wait for a new beep to start the next 30 s run. Players were told to complete as many stages as possible, and the test was ended when the players could not maintain the required running speed or could not reach the 3-m zone before the beep on three consecutive times. The test final outcomes were HR_max_ (bpm) and the V_IFT_ (km·h^−1^) score, which was determined by the final velocity reached in the last running lap [20].

#### 2.3.2. Vameval Test

The Vameval is a cardiorespiratory fitness test that consists in a progressive incremental running until exhaustion. The athletes were required to run in a circular setup with 31.85-m radius with cones placed every 20-m. The test started at 8 km·h^−1^ followed by 0.5 km·h^−1^ increments every minute. After the start, the athletes had to maintain the correct running pace as indicated by the audio recording, so that they were in line with each of the placed cones when the beep sounded. If athletes were 1-m behind a cone when a beep sounded, they were given one fault. At the second warning the test stopped. The test final outcomes were the total time in minutes and seconds to complete the test, and MAS (km·h^−1^). To calculate MAS, firstly an VO_2_max estimate was calculated (3.5 × velocity of the last lap). Then MAS was calculated as the estimated VO_2_max divided by 3.5 [21]. The V_vameval_ (km·h^−1^) was determined by the final velocity reached in the last running lap.

#### 2.3.3. The Sprinting Tests

To measure sprint performance, the 20-m sprint test, including the 5-m, 10-m and 15-m split times, was conducted. To assess sprinting times and split times, two pairs of timing gates (Smart Speed, Fusion Sport, Queensland, Australia) were used. Before the test started, a standardized sprint specific warm up was completed. A 20-m straight line was marked by a cone at the beginning (0-m) and at the end (20-m) of the space outlined for the test. The athletes started from a static position with one foot in front of the other, with the front foot behind the starting line. The athletes were instructed to start the test after a “3,2,1, go” verbal signal was made. After the signal was made, the athletes had to maximally accelerate and reach the ending line as fast as possible. Each athlete completed three 20-m sprint trials interspersed with 3 min of rest. The total time in seconds to complete each 20-m sprint was recorded. 

#### 2.3.4. Countermovement Jump

For the CMJ free arms was used allowing them to do arm swings [22]. The CMJ tests were performed on a force plate (Force Decks v1.2.6109, Vald Performance, Albion, Australia). Players were told to start from a standing position and were allowed to do a knee flexion at their comfortable depth before the jump take off. During the flight phase, the athletes had to maintain hip, knee and ankle extension and jump as high as possible. Also, players were instructed to try to land with the tip toes in the same place they took off. Three maximal trials were made and the jump heights (cm) were registered for further analysis.

### 2.4. Training Load Monitoring

#### 2.4.1. Internal Load

For the internal load objective measures, HR data were recorded using Bluetooth HR sensors (Polar H10, Polar-Electro, Kempele, Finland, recorded in 5-s intervals) synchronized to a portable 10-Hz VX Sport 350 GPS units (VX Sport, Wellington, New Zealand). HR measures including HR_avg_, Edwards’TRIMP, and Bannister’TRIMP were analyzed following each session. The HR_max_ of each individual was extracted from maximal field-based test 30–15 Intermittent Fitness Test. The test seems to be valid for extracting the HR_max_ [23]. For internal load subjective measures, approximately 30 min after each training session the Foster’s 10-point scale of the rate of perceived exertion (RPE) was applied [24]. The athletes were asked about how hard the training session was, and they had to score from 1 to 10, were 1 corresponds to “very light activity” and 10 corresponds to “maximal exertion”. The athletes scored the RPE individually without the presence of other athletes. Moreover, the athletes rated their perceived level of exertion separately for respiratory (sRPE[R]) and leg musculature (sRPE[M]) efforts, as previously detailed [10,11]. Also, they were allowed to score the RPE in decimals (e.g., 1.5). The subjective internal load was then obtained by multiplying each athlete’s RPE score by the total duration of the soccer training session in minutes to determine the session-RPE (sRPE), expressed in arbitrary units (A.U.) [25].

#### 2.4.2. External Load

External load measures were recorded during all sessions using portable 10-Hz VX Sport GPS units (VX Sport), which was previously considered a valid and reliable GPS device [26]. The external load measures included in this study were total distance (TD), high-speed running (HSR, distance >19.8 km·h^−1^), sprint distance (SD, ≥25.2 km·h^−1^) and mechanical work (MW) that summed the numbers of acceleration and deceleration efforts above and below 2.2 m·s^2^ thresholds.

### 2.5. Statistical Analysis

The results are presented in text, table and figures as Mean ± SD or 90% confidence intervals (CI) where specified. Normality of the sample and homogeneity was preliminary tested and confirmed in the Kolmogorov-Smirnov and Leven’s test, respectively (*p* > 0.05). Within-group changes in changes of V_IFT_, V_vameval_, 20-m sprint and CMJ were expressed as percentage changes and standardized differences as Cohen’s d (effect size, ES, 90% CI) [27]. No missing data occur in within group analysis. Between-group differences in changes of V_IFT_, V_vameval_ tests was also expressed based on Cohen’s d (effect size, ES, 90% CI) [27]. Magnitude-based inference approach was used for interpreting data [28]. Threshold values for ES were <0.2: trivial; 0.20–0.59: small; 0.60–1.19: moderate; >1.2: large [28]. Probabilities were calculated to indicate whether the true change was lower than, similar to, or higher than the smallest worthwhile change (SWC) [29]. The scale of probabilities was as follows: 25–75%: possible; 75–95%: likely; 95–99%: very likely; >99%: almost certain [29]. The probabilities were used to make a qualitative probabilistic mechanistic inference about the true effect: if the probabilities of the effect being substantially positive and negative were both >5%, the effect was reported as unclear; the effect was otherwise clear and reported as the magnitude of the observed value. Person correlation coefficient was also used to measure the association between V_IFT_ and V_Vameval_ outcomes as well as their changes (i.e., ∆V_IFT_ and ∆V_vameval_) with accumulated training load indices. The correlation coefficient (r, 90% confidence limits, CL) was ranked as trivial (<0.1), small (0.1–0.29), moderate (0.3–0.49), large (0.5–0.69), very large (0.7–0.89) and nearly perfect (0.9–0.99) [28]. The statistical procedures were conducted in propre-designed Excel spreadsheets [30].

## 3. Results

The results showed almost certainly moderate changes in VIFT, V_Vameval_, and CMJ following training intervention (Table 1). An almost certain large improvement was also observed in 20-m sprint in players (Table 1).

**Figure 1:**
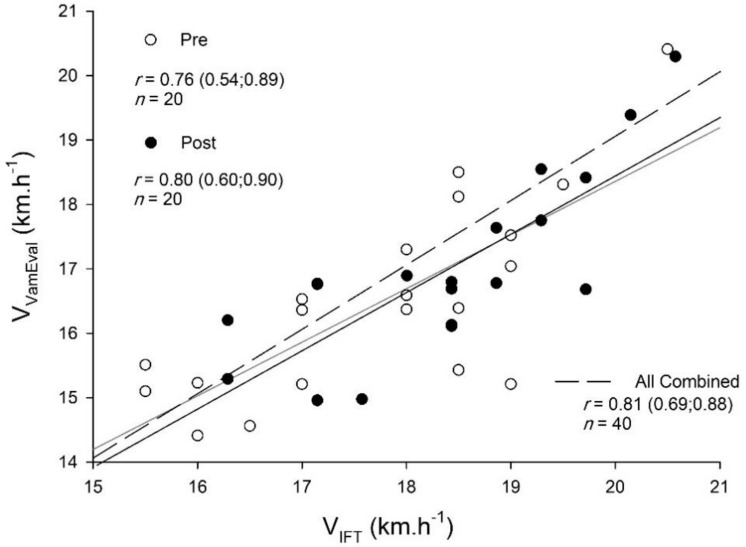
Relationships between V_IFT_ and V_Vameval_ tests for pre−, post− and pooled-data.

Very large relationships were observed between changes V_IFT_ and V_Vameval_ for pre-, post- and pooled-data (Figure 1).

**Figure 2:**
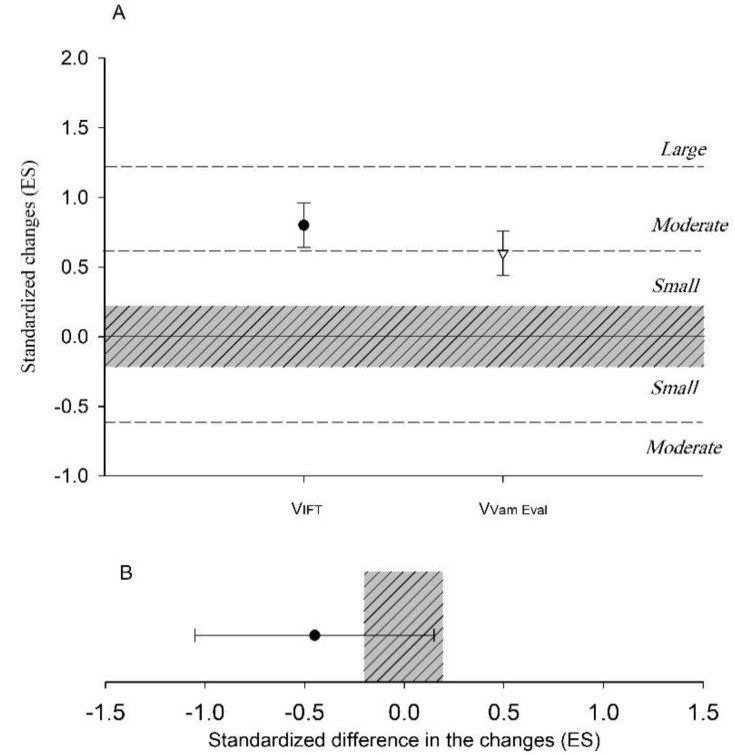
Relationships and sensitivities of V_IFT_ and V_Vameval_ to training. (**A**) Within-group changes and (**B**) between-group changes in V_IFT_ and V_Vameval_.

V_Vameval_ showed likely smaller changes (i.e., sensitivity) (−22.4%, [−45.0 to 9.4]), ES −0.45 [−1.05 to 0.16]) compared to VIFT following training intervention (Figure 2).

**Figure 3:**
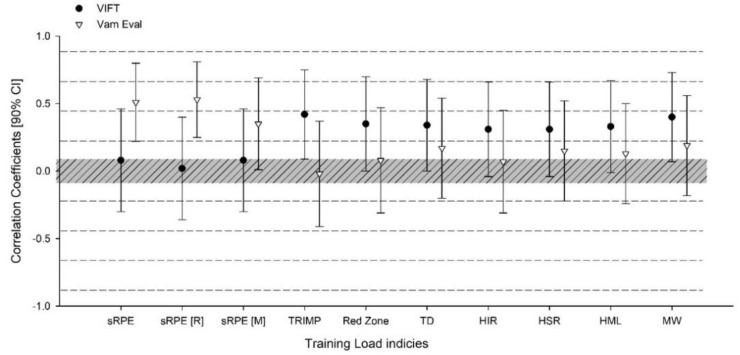
Dose-response relationships between V_IFT_ and V_Vameval._ with selected training load measures. Notes: sRPE; session-ratings of perceived exertion, sRPE (R); respiratory session-ratings of perceived exertion, sRPE (M); muscular session-ratings of perceived exertion, TRIMP; training impulse, Red Zone; time spent >85% of HR_max_, TD; total distance, HIR; high-intensity running, HSR; high-speed running, HML; high-metabolic load, MW; mechanical work (number of accelerations and decelerations >3 m·s^2^).

When analyzing dose-response relationships between ∆V_IFT_ and ∆V_Vameval_ and training load indices, ∆V_IFT_ revealed trivial unclear associations with sRPE and its differential versions (i.e., respiratory and muscular sRPE) but showed moderate correlations with all other selected measures (Figure 3). In contrast, V_Vameval_ showed large to very large relationships with sRPE and its differential versions but revealed unclear trivial-to-small associations with all other selected training load measures (Figure 3).

## 4. Discussion

The aims of the present study were to analyze the within-group changes of V_IFT_, V_Vameval_, 20-m sprint and CMJ after training intervention, and to explore the relationships between V_IFT_ and V_Vameval_ tests, and their changes, with accumulated training load indices. The main findings revealed that although there were very large relationships between changes of V_IFT_ and V_Vameval_ measures, the V_IFT_ was the most sensitive to track small changes. While V_IFT_ showed unclear associations with both sRPE measures, it showed moderate relationships with all external and objective internal loads. On the other hand, V_Vameval_ showed large-to-very large associations with both sRPE measures, but not with any external and objective internal loads.

Within-group changes (pre-post) of V_IFT_, V_Vameval_, and CMJ showed almost certain moderate improvements, and almost certainly large improvements for the 20-m sprint test were revealed after training intervention. This finding may be related to the fact that the analyzed training intervention period was from the beginning until the end of the preparation phase where greater training volumes usually ensured. In fact, some studies suggest that the overall physical fitness parameters tend to increase after the pre-season period [31,32]. However, some caution should be given to this, as some controversies has been documented regarding this topic. For instance, in contrast with our findings, no significant changes for sprint performance were found after the pre-season period [33]. Indeed, other study conducted on 19 professional Spanish soccer players revealed no significant differences for CMJ performance from the beginning of pre-season to 4-weeks after the beginning of in-season [9]. However, that study revealed significant sprint time improvements, similar to our results [9]. These setbacks may be related to the different methodologies used, considering the type of population, observational periods and the testing protocols used.

The V_IFT_ and V_Vameval_ measures showed very large associations between them, with V_IFT_ being the most sensitive measure. Previous research has documented similar associations between the 30-15IFT test and the Yo-Yo Intermittent Recovery test (YYIRT), with similar sensitivity [34]. The greater sensitivity of V_IFT_ to track small changes may be due to the fact that the 30-15IFT is more dependent to aerobic power, while the Vameval test is more related to aerobic endurance. Also, the Vameval test is done in a circular fashion, while the 30-15IFT characteristics and the test final outcome (V_IFT_) are more soccer specific [35]. For those reasons, using the 30-15IFT and V_IFT_, seem to be more useful for tracking even the smallest but worthwhile changes in performance than the Vameval test and its related V_Vameval_.

Surprisingly, the dose-response relationships between V_IFT_ and V_Vameval_ changes and internal and external loads revealed to be somewhat complex. Although meaningful correlations between 30-15IFT and Vameval tests, it seems that they might have different dose-response relationships with training loads. In the present study, only V_Vameval_ presented strong associations with all sRPE measures, while V_IFT_ showed no relationships with any of the internal subjective measures. In contrast, a study conducted on twelve professional soccer players, revealed that soccer practice volume and the accumulated subjective measures of training loads had strong associations with increases in higher velocities completed during the 30-15IFT [13]. Interestingly, it was previously revealed no relationships between sRPE, sRPE [R] and sRPE [M] with changes in aerobic fitness [9].

As mentioned before, contradictory evidence has been documented regarding the relationships between objective internal load measures and changes in field tests performances [13,14]. In fact, no relationships were found between HR measures and changes in 30-15IFT test performance, which is in contrast with our findings [13]. Conversely, other study conducted on eighteen professional soccer players, revealed large-to-very large associations between TRIMP and the yo-yo intermittent recovery 1 test performance [14]. Similarly to our results, Rabbani et al. [19] revealed that both Bannister’s and Edward’s TRIMP showed large relationships with changes at the final speed reached on 30-15IFT. However, methodological differences between the above-mentioned studies must be highlighted, as some used field tests and others used laboratory tests.

Moreover, our study demonstrated moderate relationships between V_IFT_ and all external loads, while V_Vameval_ did not show any associations. Similar to our results, a recent study conducted on 11 professional soccer players, revealed large dose-response relationships between NBL and changes in high-intensity intermittent running capacity assessed via 30-15IFT and its related V_IFT_ [19]. Although that same study [19], revealed no associations between TD and high-intensity running (HIR), and very high-intensity running (VHIR) metrics with changes in V_IFT_, which is in contrast with our results. Also, other study [8] revealed a lack of relationships between the overall external metrics and changes in aerobic fitness performance.

These differences might be related to the fact that the two above-mentioned studies [8,19], analyzed the dose-relationships during in-season period, while in the present study our observations were from pre-season period to only 4 weeks of the beginning of the season. However, it should be noted that only moderate relationships were found between V_IFT_ and all external loads. Also, MW was the metric the strongest associations compared to all other external loads, reinforcing the statement regarding the usefulness of HR-based and MW-based metrics for tracking changes in high-intensity intermittent running performance in professional soccer players [19].

The present study had its limitations. One of the main limitations is related to the small sample size, which makes any generalizations based on the results difficult. However, considering that the sample is from professional players, is almost impossible to have larger samples. Other study limitation can be related with variation in environmental conditions occurred during the period of intervention. Another limitation is the fact that only a brief period during the initial phase of the season was analyzed. These limitations may influence the results, since no dose-response relationship was analyzed in the later stage of the season, and possibly different results can be found. Future studies should analyze a more longitudinal period to make better generalizations.

Considering the lack of studies and the conflicting evidence surrounding the dose-relationships between internal and external loads with physical fitness changes, the present study revealed some interesting findings. As practical implications, this study suggests that despite field tests may show relationships between them, it does not mean that they present similar dose-response relationships with internal and external training loads. Therefore, different dimensions of load may produce different impact in fitness of players. However, conclusions should be interpreted carefully, since the limitations and small sample may affect the generalizability of the findings.

## 5. Conclusions

The present study revealed significant improvements of V_IFT_, V_Vameval_, CMJ and 20-m sprint tests after the training intervention. Despite very large relationships between V_IFT_ and V_Vameval_, the V_IFT_ showed great sensitivity to track small changes. Also, these two measures revealed dose-response relationships with different dimensions of training load quantification. Objective internal and external load measures seem to be better suited for tracking V_IFT_ changes. While subjective internal loads are better suited to track changes in aerobic endurance from V_Vameval_, at least during the initial phases of the season. For those reasons, coaches and practitioners should use objective internal and external loads to track aerobic power related changes from V_IFT_.

## Figures and Tables

**Table 1:** Within-group changes in physical fitness tests.

Group	Pre	Post	% Difference (90% CL)	Standardized Difference	% Greater/Similar/Lower
				(90% CL)	(90% CL)
				Rating	Probability
V_IFT_ (km.h^−1^)	17.8 (1.4)	19.0 (1.4)	6.8 (5.4; 8.2)	0.8 (0.64; 0.96)	100/0/0
				Moderate	Almost certain
V_Vameval_ (km.h^−1^)	16.5 (1.5)	17.5 (1.5)	5.7 (4.2; 7.2)	0.6 (0.4; 0.7)	100/0/0
				Moderate	Almost certain
20-m sprint (s)	3.0 (0.0)	2.9 (0.0)	−2.8 (−3.9; −1.6)	−1.1 (−0.6; −0.6)	0/0/100
				Large	Almost certain
CMJ (cm)	46.7 (3.3)	49.7 (3.9)	5.5 (3.9; 7.2)	0.7 (0.5; 0.9)	100/0/0
				Moderate	Almost certain

V_IFT_: The maximal speed reached at the end of 30–15 Intermittent Fitness Test, Vameval: The maximal speed reached at the end of the Vameval test, CMJ: Countermovement jump, CL: Confidence limits.
